# Factors associated with maternal mortality in Sub-Saharan Africa: an ecological study

**DOI:** 10.1186/1471-2458-9-462

**Published:** 2009-12-14

**Authors:** Jose Luis Alvarez, Ruth Gil, Valentín Hernández, Angel Gil

**Affiliations:** 1 Area of Preventive Medicine and Public Health, University Rey Juan Carlos, Madrid, Spain

## Abstract

**Background:**

Maternal health is one of the major worldwide health challenges. Currently, the unacceptably high levels of maternal mortality are a common subject in global health and development discussions. Although some countries have made remarkable progress, half of the maternal deaths in the world still take place in Sub-Saharan Africa where little or no progress has been made. There is no single simple, straightforward intervention that will significantly decrease maternal mortality alone; however, there is a consensus on the importance of a strong health system, skilled delivery attendants, and women's rights for maternal health. Our objective was to describe and determine different factors associated with the maternal mortality ratio in Sub-Saharan countries.

**Methods:**

An ecological multi-group study compared variables between many countries in Sub-Saharan Africa using data collected between 1997 and 2006. The dependent variable was the maternal mortality ratio, and Health care system-related, educational and economic indicators were the independent variables. Information sources included the WHO, World Bank, UNICEF and UNDP.

**Results:**

Maternal mortality ratio values in Sub-Saharan Africa were demonstrated to be high and vary enormously among countries. A relationship between the maternal mortality ratio and some educational, sanitary and economic factors was observed. There was an inverse and significant correlation of the maternal mortality ratio with prenatal care coverage, births assisted by skilled health personnel, access to an improved water source, adult literacy rate, primary female enrolment rate, education index, the Gross National Income per capita and the per-capita government expenditure on health.

**Conclusions:**

Education and an effective and efficient health system, especially during pregnancy and delivery, are strongly related to maternal death. Also, macro-economic factors are related and could be influencing the others.

## Background

It is estimated that more than 500 000 women die every year in the world due to complications related to pregnancy or childbirth; half of them live in Sub-Saharan Africa (SSA) [1]. However, among all the regions of the world, SSA (where the problem is most acute) bears the lowest annual reduction rate: 0.1% [2]. In fact, the overall number of maternal deaths has increased between 1990 and 2005.

The WHO (World Health Organization) calculates a global ratio of 400 maternal deaths per 100 000 live births [3]. Where nothing is done to avoid maternal death, "natural" mortality is around 1000-1500 per 100 000 births (an estimate based on historical studies and data from contemporary religious groups who do not intervene in childbirth). Thanks to multiple worldwide efforts from health services and medical interventions [4], the maternal death ratio is currently four times smaller than its "natural" levels. Nearly all natural maternal deaths are prevented in developed countries (only 1% of maternal deaths occur in the developed world), while only one third of these deaths are prevented in African countries (Sierra Leone and Niger are over this figure). Of the 20 countries with the highest maternal mortality ratios, 19 are in sub-Saharan Africa; only Afghanistan is not in this region. Additionally, maternal deaths are more inequitably spread across the globe than newborn or child deaths, and maternal mortality reflects the status of women and is a sensitive indicator of inequality [5].

Maternal deaths result from a wide range of indirect and direct causes. The major direct causes in Africa are haemorrhage (34%), infection (10%), hypertensive disorders (9%) and obstructed labour (4%). This is different from the developed countries where haemorrhage only accounts for 13% and the primary cause of maternal mortality is hypertensive disorders such as eclampsia [6]. Indirect causes represent 20% of the total; they are pre-existing or concurrent diseases that are not complications of pregnancy, but that are complicated during pregnancy or aggravated by it [6].

With regard to the timing of maternal death, between 11% and 17% of maternal deaths happen during childbirth itself and between 50% and 71% occur in the postpartum period [7]. The fact that a high level of risk is concentrated during childbirth itself and that many postpartum deaths are also a result of what happened during birth shows that it is important to give attention to mothers during the hours, and sometimes days, that are spent in labour and giving birth. These are the critical moments when a joyful event can suddenly turn into an unforeseen crisis.

However, mortality only shows a portion of the global suffering because for each death it is estimated that 20 more women will live with lasting sequelae. These are even more difficult to quantify than maternal deaths [8].

Most of the deaths and disabilities attributable to childbirth are avoidable because the medical solutions are well known. The challenge that remains is therefore not technological but strategic and organisational. The millennium declaration of the United Nations was approved in 2000 by 189 States and includes 2 goals related to Maternal Health: reduce the maternal mortality ratio by three-quarters between 1990 and 2015 and achieve universal access to reproductive health by 2015 [9]. Achieving this Fifth Millennium Development Goal (MDG 5) will require political participation, resources and suitable strategies. Some studies have searched for the determinants of maternal mortality in developing countries [10,11], but a review of the latest data shows that taking into account other variables not often assessed in these studies, especially educational variables which are often oblivious, is needed if we aim to determine if MDGs are achievable.

This study was therefore proposed for the purpose of determining different types of factors (Health care system-related, educational and economic) associated with the high maternal mortality ratio in Sub-Saharan countries. The study uses statistical models and the latest data obtained from the main international information sources: WHO (World Health Organization), World Bank, UNICEF (United Nations Children's Fund), and UNDP (United Nations Development Programme).

## Methods

### Study design and population

An analytical ecological, multi-group study was designed to compare the Maternal Mortality Ratio (MMR) between SSA countries during the period between 1997 and 2006 and to assess the health care system, economic and developmental indicators related to the variability of MMR between countries.

Each SSA country was used as an analysis unit. The selected SSA countries were those indicated by the WHO as African region countries, with the exception of Algeria (considered a Maghreb Country), resulting in a total of 45 countries: Angola, Benin, Botswana, Burkina Faso, Burundi, Cameroon, Cape Verde, Central African Republic, Chad, Comoros, Congo, Congo-Democratic Republic, Côte d'Ivoire, Equatorial Guinea, Eritrea, Ethiopia, Gabon, Gambia, Ghana, Guinea, Guinea-Bissau, Kenya, Lesotho, Liberia, Madagascar, Malawi, Mali, Mauritania, Mauritius, Mozambique, Namibia, Niger, Nigeria, Rwanda, Sao Tome and Principe, Senegal, Seychelles, Sierra Leone, South Africa, Swaziland, Tanzania, Togo, Uganda, Zambia and Zimbabwe. For descriptive purposes, countries were grouped depending on their Human Development Index (HDI) as high, medium and low HDI according to UNDP data [12].

### Study variables

The information sources were the latest available data from the WHO [13], UNICEF [14], UNDP [15] and World Bank [16] (between 1997 and 2006). Population data was obtained from the World Bank. We carried out a secondary analysis of these existing databases. The result variable was the MMR per 100 000 live births; this ratio is the number of deaths among women from any cause related to or aggravated by pregnancy or its management (excluding accidental or incidental causes) during pregnancy, childbirth, or within 42 days of termination of pregnancy, irrespective of the duration or site of the pregnancy, for every 100 000 live births in a given year or period of time [13].

Indicators providing insight as to how the health services in the country operate were used as the determinant variables. The following Health care system-related variables that show health status and health access were selected: infant mortality rate [13], antenatal care coverage [14], births attended by skilled health personnel [15], proportion of the population with sustainable access to an improved water source [13] and proportion of the population with access to improved sanitation [13]. Additionally, the following educational variables, which show educational progress and instruction, were selected: adult literacy rate [13], contraceptive prevalence rate [15] (contraceptive rates are more related to cultural, educational, and social factors rather than to health status), ratio of female literacy rate to male rate [15], net primary enrolment rate [15], net primary enrolment rate female [15] and the education index [15]. Others variables selected were economic: public expenditure on health [16], public expenditure on education [16], gross national income per capita [16], external resources for health as a percentage of total expenditure on health [13], government expenditure on health as a percentage of the general government expenditure on health [13], out of pocket expenditure as a percentage of private expenditure on health [13] and per-capita government expenditure on health [13]. These variables were selected because of the evidence existing in previous studies about their relationship with MMR [17]. They were grouped in 3 categories according to their definitions. The variables and their definitions are shown in Table 1.

**Table 1:** Description of variables

Variable	Description
HEALTH CARE SYSTEM-RELATED	

Infant mortality rate	Number of deaths among infants (<1 year of age) per 1000 live births in a given year
Antenatal care coverage	Percentage of women aged 15-49 years that were attended at least once during pregnancy by skilled health personnel (doctor, nurse, or midwife)
Births attended by skilled health personnel	Percentage of births that received care from qualified medical personnel
Access to an improved water source	Percentage of the population who use any of the following types of water supply for drinking: piped water, public tap, borehole or pump, protected well, protected spring or rainwater
Access to improved sanitation	Percentage of the population with access to facilities that hygienically separate human excreta from human, animal and insect contact. Facilities such as sewers or septic tanks, pour-flush latrines and simple pit or ventilated improved pit latrines are assumed to be adequate, provided that they are not public
EDUCATIONAL	

Adult literacy rate	Percentage of persons aged 15 years and over who can both read and write
Contraceptive prevalence	Percentage of married women aged 15-49 that use contraceptive measures.
Ratio of female literacy rate to male rate	The data refer to national literacy estimates from censuses or surveys
Primary enrolment rate	Number of pupils of the theoretical school-age group who are enrolled in primary school expressed as a percentage of the total population in that age group
Primary enrolment rate, female	The same as the net primary enrolment rate, but only for the female group
Education index	This measures a country's relative achievement in both adult literacy and combined primary, secondary and tertiary education.
ECONOMICAL	

Public expenditure on health	This measures the final consumption of health care goods and services as percentage of the GNP
Public expenditure on education	This measures the final consumption of education as percentage of the GNP
GNI per capita	Gross National Income (in current US Dollars) divided by midyear population. The atlas method is used
External resources for health	This includes all grants and loans for health goods and services passing through governments or private entities, in cash or in kind as a percentage of total expenditure on health
Government expenditure on health	This gives an idea as to the priority that the health issues are given in government budgets expressed as percentage of the general government expenditure on health
Out-of-pocket expenditure on health	These are the direct outlays of households, including gratuities and in-kind payments made to health practitioners, pharmaceutical suppliers, therapeutic appliances, and other goods and services, as a percentage of the private expenditure on health.
Per-capita government expenditure on health	This is the government expenditure on health per person

### Statistical analysis

Based on the valid data obtained, we performed a descriptive analysis of both the independent and dependent variables of interest by using centralisation and dispersion measures. The normality of the quantitative variables was verified using the Kolmogorov-Smirnov test. In order to reach normality, log transformation was used for the non-normal variables. Where variables did not follow a normal distribution, non-parametric tests were used. Once this was checked, a Pearson/Spearman correlation analysis was performed to ascertain the degree of the relation between MMR and the remaining variables.

All hypothesis testing to determine differences, associations and relationships was deemed significant at p < 0.05. Statistical data analyses were performed using the SPSS computer software programme (Statistical Package for Social Sciences) version 16.0.

## Results

**Figure 1:**
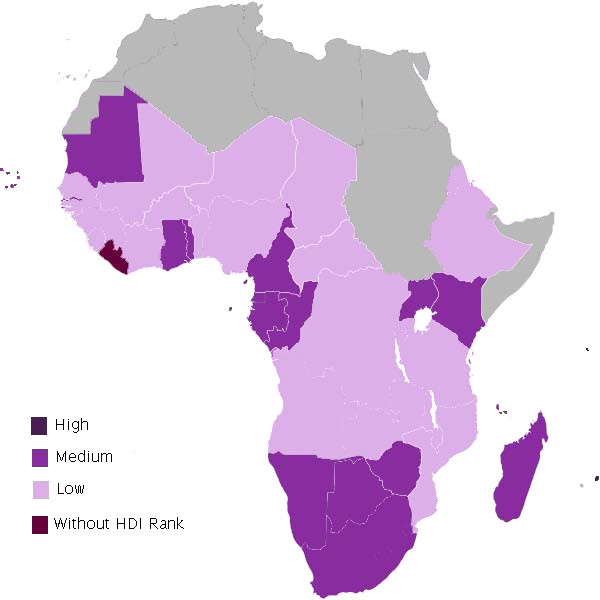
**Africa map indicating HDI (2005)**.

The descriptive analysis of the data is shown in table 2; countries appear grouped according to its Human Development Index (HDI). Just two countries of the SSA region are High HDI countries: Mauritius and Seychelles; and Liberia is without HDI rank (see Figure 1). MMR ranged from 15 deaths per 100 000 live births in the countries classified as High in the Human Development Index (HDI) to 2100 deaths per 100 000 in Sierra Leone. In spite of the important differences among these countries, this ratio was generally quite high everywhere, and the mean value was 885. The values of the Health care system-related indicators were also striking with percentages of antenatal care, births attended by health personnel, access to water and sanitation often below 50%. The educational indicators showed higher means than the other indicators, but they do not reach necessary levels. Economic variables, especially GNI per capita, were far below those of developed countries.

**Table 2:** Maternal Mortality Ratio and Health care system-related, educational and economical indicators of SSA countries: mean, SD and ranges for countries grouped according to its Human Development Index (HDI)

			HDI COUNTRIES		
			
Indicator	MEAN	SD*	HIGH	MEDIUM	LOW
Maternal mortality (deaths per 100 000 live births)	885	321	15	210-1000	450-2100

HEALTH CARE SYSTEM-RELATED					

Infant mortality (deaths per 1000 live births)	97	24	12-13	26-123	50-165

Antenatal care coverage (%)	71	20	-	64-99	28-94

Births attended by skilled health personnel (%)	44	21	98	39-94	6-68

Access to an improved water source (%)	56	17	88-100	43-95	22-84

Access to improved sanitation (%)	37	15	-	18-65	9-61

EDUCATIONAL					

Adult literacy rate (%)	58	18	84-91	51-89	23-73

Contraceptive prevalence (%)	23	14	76	18-60	3-34

Ratio of female rate to male rate (%)	0.70	0.17	<1**	<1**	<1**

Primary enrolment rate (%)	71	13	95-99%	44-97	40-95

Education index (%)	0.57	0.14	0.81-0.88	0.45-0.81	0.25-0.66

ECONOMICAL					

Public expenditure on health (%GNP)	2.2	1.3	2.4-4.6	1.1-9.9	0.7-9.6

Public expenditure on education (%GNP)	4.5	1.7	4.5-5.4	0.6-13.4	2-6.1

Gross National Income per capita ($)	586	804	4081-7547	100-3865	110-647

External resources for Health (%)	16	11	1.4-4.6	0.4-45.4	1.4-49

Government expenditure on Health (%)	7.9	3.4	8.7-9	4.2-13.8	2-16

Out of pocket expenditure on Health (%)	78	23	62-77	17-100	39-100

Per-capita government expenditure on health ($)	34	65	95-346	3-192	1-19

*SD: standard deviation

** The ratio of female literacy rate to male literacy rate was far below 1 with the exception of Seychelles (1.01), Botswana (1.02) and Lesotho (1.23).

The Kolmogorov-Smirnov test showed that all variables had normal distribution with the exceptions of Gross National Income per capita and per-capita government expenditure on health. Non-parametric tests were used for these two non-normal variables.

Table 3 shows the correlation between the MMR and the remaining variables. In the case of Health care system-related indicators, a strong, direct relationship between the MMR and the infant mortality rate was observed; a strong, inverse relationship with prenatal care coverage, births assisted by a skilled health personnel, and access to an improved water source was also observed. In relation to economic indicators, no significant relationship was observed with the exception of the Gross National Income per capita, the per-capita government expenditure on health and the out of pocket expenditure on health. The educational indicators showed inverse relationships with the MMR in all cases and were significant and strong for adult literacy rate, contraceptive prevalence, ratio of female rate to male rate, primary enrolment rate, primary female enrolment rate and education index.

**Table 3:** Correlation between Maternal Mortality Ratio and country indicators

Indicator	N	r*	p
Maternal mortality (deaths per 100 000 live births)	43	1	

HEALTH CARE SYSTEM-RELATED			

Infant mortality (deaths per 1000 live births)	43	0,815	< 0,001

Antenatal care coverage (%)	42	-0,413	0,006

Births attended by skilled health personnel (%)	43	-0,572	< 0,001

Access to an improved water source (%)	43	-0,399	0,008

Access to improved sanitation (%)	43	-0,261	0,091

EDUCATIONAL			

Adult literacy rate (%)	39	-0,517	< 0,001

Contraceptive prevalence (%)	42	-0,622	< 0,001

Ratio of female rate to male rate (%)	39	-0,485	0,002

Primary enrolment rate (%)	37	-0,386	0,018

Primary enrolment rate, female (%)	33	-0,435	0,012

Education index (%)	43	-0,534	< 0,001

ECONOMICAL			

Public expenditure on health (%GNP)	43	-0,109	0,488

Public expenditure on education (%GNP)	39	-0,242	0,138

Gross National Income per capita ($)	43	-0,456	0,002

External resources for Health (%)	43	0,266	0,085

Government expenditure on Health (%)	43	-0,12	0,442

Out-of-pocket expenditure on Health (%)	43	0,365	0,016

Per-capita government expenditure on health ($)	43	-0,452	0,002

* r: correlation coefficient from Pearson or Spearman tests.

## Discussion

In spite of the efforts in many sub-Saharan countries to reduce the maternal mortality ratio in recent decades, it shows no evidence of decline. This study takes into account several factors reflecting aspects of women's status to predict maternal mortality. We found a positive relationship not only with the health care system-related variables as other studies have shown [18], but also with educational and economic variables where attention should be focused.

Before assessing these data, it is necessary to keep in mind that the data have been obtained from four different databases: WHO, World Bank, UNICEF and UNDP. As their data collection methodology is similar, the effect on the final result is probably not relevant. It is also important to know that in some cases the information sources are based on estimates; however, the existing data provide an approximate idea as to what happens in these countries. A first step can be taken with the existing data to plan future studies in this area. It is often said that it is necessary to improve information and recording systems in SSA countries [19]. However, it cannot be improved if the problems and actual difficulties are not known. In addition, other important variables such as political instability, poverty and corruption are related with sanitary indicators and are difficult to collect. Grouping the variables into educational, economics and health care system-related variables may be arbitrary and correlations between variables and categories can be present. As we are performing an ecological study, cause-effect relationship cannot be confirmed and conclusions cannot be inferred to individual subjects [20].

The MMR in SSA for 2005 is estimated at 835 deaths per 100 000 live births (IC 95% 300-1400). This is striking because of its high value (Table 2) as well as its considerable deviation (15-2100). This shows that there are significant differences among the different countries even though this rate is generally much higher than on any other continent [5]. Antenatal care coverage and medical professionals attending births are two measures that are always recognised to be crucial in reducing the MMR [21]; we often find figures in SSA that are between 50% and 80% for these indicators. The lack of basic infrastructure (water and sanitation) and the lack of basic formal education are factors associated with the infant mortality rate [22], which is shown through bivariate analysis to correlate to the MMR. The economic indicators also show marked differences compared with developed countries in absolute terms. In addition, the percentage of the GNP devoted to education and health are different from those of the first world. For example, the percentage of GNP devoted to education and health in the USA is 5.9 and 6.9, respectively; in France, these percentages are 5.9 and 8.2, respectively [15].

The bivariate analysis demonstrated the relationship between several factors and the MMR. The strong and direct correlation between the MMR and the infant mortality rate was striking but maybe not surprising given that in SSA mothers are a fundamental basis for the survival of their children. The inverse correlation with the adult literacy rate and the rate of enrolment in primary school was also very strong. The efforts of many organisations to provide basic education, especially for mothers, give SSA countries a more hopeful future because a higher education reduces the MMR, morbidity and misuse of health services [23]. Economic factors such as the Gross National Income per capita and per-capita government expenditure on health showed an inverse correlation with the MMR. The out-of-pocket expenditure on health presented a significant direct correlation to the MMR: the more out-of-pocket expenditure in health in a country the higher the MMR. This could support what has been published in relation to the financial system inequity and inefficacy [24]. The money families need to spend from their own pockets in order to obtain health shows that the Health System is not working properly. We also observed (table 2) a relationship between the MMR and the HDI [25].

All the three groups of variables, educational, economic and health care system-related variables, are interrelated and thus it is difficult to tell which of these variables is the strongest determinant. Our findings might point to an important macro-economic factor related to the educational and health care system-related factors that ultimately influences the MMR. Table 3 shows that all these factors are strongly correlated and interventions must be aimed at all these groups together if we aim to reduce maternal mortality.

The state of women's health in SSA today can be described through statistics that show death, disability and suffering. Technical interventions can be identified that could prevent or treat the majority of conditions that kill women of reproductive age, and thus enable all countries to meet the Millennium Development Goals,. Our study shows that important efforts should be made to strengthen the sanitary system and focus on delivery, the postpartum period, obstetric care [26], and those educational factors pertaining to development as well as the economic factors that are interrelated. It is not only supply-side factors (the availability of high-quality services), but also demand-side factors (educational factors to appropriate utilisation) that are relevant. For example, complications of unsafe abortion, which now account for some 13% of maternal deaths globally, could also be prevented through access to contraception and safe abortion services [27]. It is clear that the Millennium Goals will not be met with an annual per capital health expenditure of $5-$10 or less. However, even massive amounts of new aid given to help the same old strategies will not lead to success.

## Conclusions

In conclusion, education, especially the education of women, is higher in those countries were the MMR is lower and increases the general health of the population, as has been observed in the example of Kerala and Neuquén [28]. Additionally, an effective and efficient health system, especially during pregnancy and delivery, and access to safe drinking water are fundamental pillars for maternal health. Economic factors are related to the aforementioned factors and therefore will be very difficult to improve health without taking them into account. It is essential that our efforts focus on these factors if we aim to fulfil each of the Millennium Development Goals for 2015 [29].

## Competing interests

The authors declare that they have no competing interests.

## Authors' contributions

JLA acquired the data, wrote the paper and made the principal contributions to conception, design and interpretation of the data. RG was involved in drafting the manuscript and revising it critically as well as participating in the design and analysis. VH performed the statistical analysis. AG conceived the study and participated in its design and coordination and helped to draft the manuscript. All authors read and approved the final manuscript.

## Pre-publication history

The pre-publication history for this paper can be accessed here:

http://www.biomedcentral.com/1471-2458/9/462/prepub
